# Nursing Progress and Its Developments: The Local Finance Committee

**Published:** 1918-09-14

**Authors:** 


### THE MATRONS' AND SISTERS' DEPARTMENT.

### NURSING PROGRESS AND ITS DEVELOPMENTS.

### The Local Finance Committee.

THE Local Executive Committees of the College of Nursing hold all the strands relating to the business of Local Centres in their hands. They decide what persons shall be invited from time to time to sit upon the General Committee; they formulate the policy of the Centre, develop its resources, arrange and carry out a plan of action. The expenses they incur are small, chiefly stationery and postage, with the hire of rooms for meeting. These Executive Committees are not, however, and cannot as a rule be charged with the duty of enlisting financial support for the Centre. The business of raising funds with which to develop the Centres on an adequate scale must be delegated to another body. This body is a vital if not necessarily permanent part of the machinery of the Local Centre. It is the Finance Committee.

Only since the foundation of the College of Nursing have the needs of the nursing profession, their claim on the public for endowment, begun to be recognised. Even now this claim is liable to be misunderstood not only by factious people, but by some nurses themselves. The call for endowments is threefold, corresponding to the three stages of the nursing career. In the course of her study years she needs endowments for scholarships and other educational advantages. In the practice of her profession she needs facilities for recreation, the stimulus of professional meetings, a substitute, in short, for the free current of every-day life from which, by the conditions of her work, she is frequently deprived for long periods. Lastly, in sickness, in disablement, in old age, she needs an arm on which to lean. The Royal National Pension Fund for Nurses, while providing a tower of strength for those who early seek its shelter, is powerless to extend its aid to those who have neglected its offers. Other benevolent societies for nurses cover but a tiny corner of the field. Thus in this direction, as well as educationally and socially, the need for endowments is widespread and pressing.

Nursing is exacting in its demands on body and mind to an extent which absorbs at certain times the whole output of vital energy. Whether this will always hold good of the nursing profession is largely a question of successful co-operation. But that it is so at the present moment, and always has been so in the past, admits of no dispute. The main reason why nurses are not business women is that they sacrifice their own interests freely on the altar of duty. The public is beginning to discover that it is their affair to redress the balance and give back to the nurse what she has voluntarily forfeited.

It is essential to the success of a body like the Local Finance Committee, formed to invite endowments for the benefit of women doing national work of supreme importance, that it should be so constituted as to command public confidence. There is perhaps no locality in this country which does not afford conspicuous examples of public-spirited interest in hospitals and nursing. In many there exists a kind of noble competition for the honour of promoting schemes for the benefit of the sick and of those who tend them. There is little difficulty in selecting coadjutors in the task of letting the public know what is wanted and how it may best be attained. The Local Executive Committee may count on the readiness of representative men and women to lend that outside aid to the nursing profession without which their onward course must be seriously impeded. The Centres will be wise to trust this part of their development to men and women who have proved their public spirit in the past. They will thus be relieved of a burden they are themselves ill-qualified to shoulder. To welcome and turn into a fruitful channel without misgiving the enthusiasm of the public for the welfare of nurses is the plain duty of those who direct the policy of the Centre. Working in full harmony with the Executive Committee through the presence on both bodies of the Local Representative, the Finance Committee will have much to teach, the nurse members of the Centre much to learn, in the conduct of business. If it be a reproach to nurses that few of them are qualified business women, it is one which can best be wiped out by learning through association with business men in the conduct of their affairs how to rule their own house in the future.

